# Factors associated with birthweight and adverse pregnancy outcomes among children in rural Guinea-Bissau - a prospective observational study

**DOI:** 10.1186/s12889-021-11215-8

**Published:** 2021-06-17

**Authors:** Alexander Dahl Stjernholm, Sanne Marie Thysen, Igualdino Da Silva Borges, Ane Bærent Fisker

**Affiliations:** 1 OPEN, Institute of Clinical Research, University of Southern Denmark, Odense, Denmark; 2 Bandim Health Project, Bissau, Guinea-Bissau; 3 Research Center for Vitamins and Vaccines (CVIVA), Bandim Health Project, Statens Serum Institut, Copenhagen, Denmark; 4 Center for Global Health (GloHAU), Aarhus University, Aarhus, Denmark

**Keywords:** BCG vaccine, Guinea-Bissau, Birthweight, Stillbirth, Perinatal death

## Abstract

**Background:**

Low birthweight (LBW) is associated with higher mortality and morbidity, but there is limited data on the prevalence of LBW in rural Africa, where many births occur at home. The Bacillus Calmette-Guérin (BCG) vaccine has non-specific effects. Studies suggest that maternal BCG-vaccination may affect the health of the child.

**Methods:**

The present study is nested within a randomised trial in rural Guinea-Bissau: Pregnancies were registered at two-monthly village visits, where information on BCG scar status and other background factors were obtained. Children were enrolled in the trial and weighed at home within 72 h after birth. In this prospective observational study, we assessed factors associated with adverse pregnancy outcomes and birthweight in binomial and linear regression models.

**Results:**

Among 1320 women who had their BCG scar status assessed, 848 (64%) had a scar, 472 (36%) had no scar. The risk of adverse pregnancy outcomes (miscarriages, stillbirths, early neonatal deaths) tended to be higher among BCG scar-negative women (13%) than among women with a BCG scar (10%), adjusted prevalence ratio = 1.29 (0.99–1.68). Birthweight was assessed for 628 (50%) of the 1232 live born children. The mean birthweight was 2.89 kg (SD 0.43) and the proportion of LBW children was 17% (104/628). Sex, twinning, region of birth, maternal age, maternal mid-upper arm circumference (MUAC), antenatal consultations, parity and possession of a mobile phone were associated with birthweight, while maternal BCG scar status was not.

**Conclusions:**

This study provides the first birthweight data for home-born children in rural Guinea-Bissau, with a mean birthweight of 2.89 kg (SD 0.43) and a LBW prevalence of 17%. We found a tendency for higher risk of adverse pregnancy outcomes among BCG scar-negative women. Birthweight was similar in children of mothers with and without BCG scar.

**Supplementary Information:**

The online version contains supplementary material available at 10.1186/s12889-021-11215-8.

## Background

Low birthweight (LBW; < 2500 g) is associated with increased mortality, morbidity, risk of infections and other deficits [1–3]. LBW is due to either preterm birth and/or intrauterine growth restriction and may have a number of underlying causes including both maternal and foetal illness [4, 5]. The majority of LBW children are born in Asia and Africa [1]. LBW estimates carry much uncertainty due to the lack of data on birthweight, [1] as many children are born outside health facilities.

World Health Organization (WHO) recommends Bacillus Calmette-Guérin (BCG) vaccine at birth in countries with a high burden of tuberculosis (TB) [6]. While the vaccine is recommended to protect against TB, some studies have found that BCG vaccination is associated with larger reductions in mortality than can be attributed solely to protection against TB, coined non-specific effects (NSE) [7–9]. In 2014, the evidence on NSEs for BCG was reviewed by the Strategic Advisory Group of Experts on Immunization, which concluded that BCG vaccination may be associated with reduced all-cause mortality [7]. Immunological studies show that BCG can induce epigenetic changes of the innate immune system which supports that BCG may have beneficial NSEs [10, 11].

It is well established that maternal antibodies, transferred during pregnancy and through breast feeding, confer protection against infectious diseases in early life [12]. It is, however, unknown whether NSEs can be transferred from mother to child and confer additional protection and better health of the unborn child. A recent immunological study among Ugandan mother-infant pairs found that maternal BCG vaccination was associated with increased pro-inflammatory immune responses in infants after BCG vaccination [13]. A randomised trial among Danish neonates, found that the association between BCG and infectious disease hospital admission differed by maternal BCG status [14]. In a study from Guinea-Bissau, having a BCG scar was associated with 66% (95% Confidence Interval (CI) 17–67%) lower mortality among children born to women with a BCG scar. In contrast, mortality was similar for children with and without BCG scars among children born to mothers with no BCG scar [15]. Thus, maternal BCG status may influence the developing immune system, health and birthweight of the child.

In low-income countries, vaccination status of adults is often difficult to determine due to a lack of health records. However, when correctly administered, the BCG vaccine causes lifelong scarification at the injection site, making the BCG scar an easy-assessable marker for BCG vaccination [16, 17]. Among BCG-vaccinated children, development of a BCG scar is associated with lower all-cause mortality [16, 18–20] suggesting that a BCG scar is an indicator of a successful BCG vaccination with beneficial NSEs.

In this prospective observational study, we take advantage of a cohort of primarily homeborn children to report birthweight data from rural Guinea-Bissau. We investigate several background factors, including maternal BCG scar status, for their association with birthweight. As underlying poor foetal health may also affect the likelihood of being weighed, we assess whether the same background factors are associated with adverse pregnancy outcomes (miscarriages, stillbirths, and early neonatal deaths (death prior to weighing within the first 72 h of life)).

## Methods

### Setting and study population

Bandim Health Project (BHP) runs a Health and Demographic Surveillance System (HDSS) in rural Guinea-Bissau, [21]. Mobile data collection teams follow women of fertile age and children below 5 years of age through home visits. Women are registered when they reach fertile age or move into the study area. Newly registered women are interviewed about their age, obstetric history, and whether they have attended school. At all visits, the women are asked whether they are pregnant. When a pregnancy is registered, the woman’s nutritional status is assessed by measurement of her mid-upper-arm circumference (MUAC) and information on socio-economic factors (type of roofing, type of toilet, possession of a mobile phone, radio and generator) are recorded. Information on antenatal care is collected prior to giving birth, and at the first visit after delivery. At all visits, information on pregnancy outcome, and vital status of all children are collected. The present prospective observational study is nested within a cluster-randomised trial assessing the effects of early BCG and oral polio vaccine (OPV) on early infant mortality and morbidity (the BCGR trial). The trial is described in more detail elsewhere [22]. The BCGR trial was conducted in three of the nine rural health regions in Guinea-Bissau: Oio, Biombo and Cacheu and enrolments started 28 July 2016. In these three regions, village visits were conducted every 2 months.

The pregnancy registration at the two-monthly visits was complemented by continuous pregnancy registration conducted by local community key informants. The mobile data collection teams confirmed the pregnancies registered by the community key informants at the two-monthly visits and informed the women about the BCGR trial. All pregnant women were invited to a temporary health post in the village. Here a BHP nurse thoroughly explained the trial. If the woman consented to participate in the trial, the BHP nurse assessed BCG scar status of the woman by examining her arms.

### Assessment of birthweight

Following a delivery, the community key informant or the woman herself informed BHP about the birth. A nurse was then dispatched to visit the new-born child within 72 h after birth. At this visit, the nurse examined and weighed the undressed child to the nearest 50 g using a digital scale (Charder MS 4400).

### Statistical analyses

We described birthweight by calculating the mean birthweight and standard deviation (SD). We furthermore calculated the proportion of children with a birthweight below 2500 g (LBW). We assessed whether maternal BCG scar status and other available background factors for the mother (region of residence; type of roof; possession of a radio, a generator/solar panel, a mobile phone; type of toilet; years of maternal schooling; maternal age), for the pregnancy (number of prenatal consultations; maternal MUAC; total number of pregnancies), and for the delivery (sex; place of birth and season of birth) were associated with birthweight and with the risk of being LBW. We used linear regression to quantify the mean difference in birthweight and compared the proportions of LBW infants using binomial regressions with a log-link function to estimate relative difference as the prevalence ratio (PR). Since, neonatal weight is known to be very dynamic, [23] we examined whether the weight differed according to age at weighing (within the first 72 h). Five sets of twins were included in the birthweight analyses. We conducted sensitivity analyses including a) only the heaviest and b) including only the lightest twin. Since we hypothesised that maternal BCG scar status might be related to birthweight, we assessed whether the background factors were associated with the mother having a BCG scar using binomial regression with a log-link function.

**Fig. 1:**
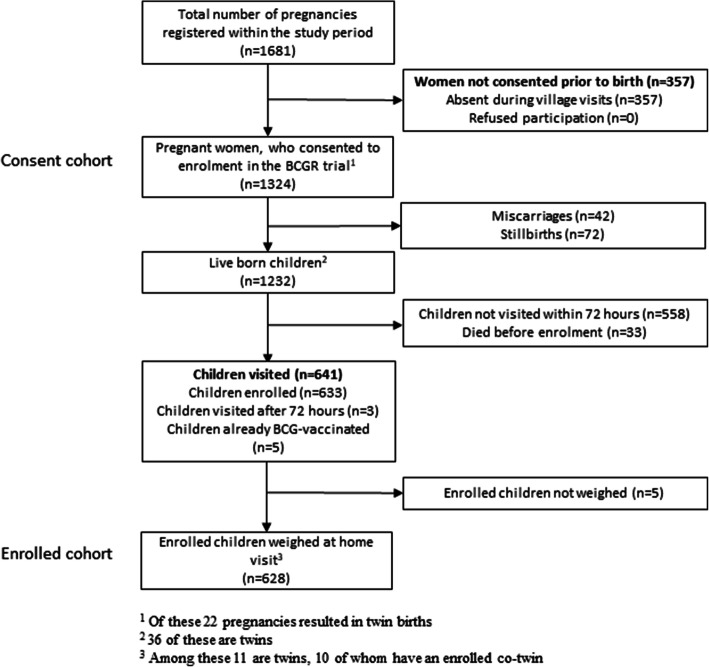
Flowchart of study participants

Birthweight was only assessed for children who were live-born and enrolled in the BCGR trial. For all pregnant women, who consented to participate in the BCGR trial (Fig. 1), we assessed the association between maternal BCG scar status and adverse pregnancy outcomes (miscarriage, stillbirth or early neonatal death prior to weighing and enrolment in the BCGR trial) using binomial regression. A twin pregnancy resulting in both a live and a stillborn child or a child who died before weighing was considered a pregnancy with an adverse pregnancy outcome. We then assessed whether associations between maternal BCG scar and adverse pregnancy outcomes or birthweight, respectively, were affected by other measured background factors by including the variables one by one. We adjusted for factors that changed the estimate by more than 5%.

If maternal BCG affects the chance of children being carried to term, a difference in gestational age could potentially affect birthweight. Time of pregnancy registration is independent of maternal BCG scar status. Thus, if having a maternal BCG scar is not associated with the risk of preterm delivery, intervals between time of pregnancy registration and delivery should be independent of having a BCG scar. We therefore used differences in time from registration to delivery as a proxy for differences in gestational age, and assessed, whether maternal BCG scar status was associated with time between pregnancy registration and delivery, using linear regression.

We used robust standard errors in all analyses to account for clustering of observations within village clusters. Analyses were performed using Stata® version 14.1.

## Results

Between November 7th, 2015 and November 2nd, 2017, 1324 women with pregnancies ending after 28 July 2016 consented to participate in the BCGR trial and 633 children were enrolled (Fig. 1).

### Determinants of birthweight

Birthweight was assessed for 628 children, of these 67% were home born. The mean birthweight was 2.89 kg (SD 0.43) and 104 children (17%) were LBW (Table 1). Girls had lower birthweight than boys and twins had lower birthweight than singletons. Children in the region of Oio had the lowest mean birthweight and considerably higher risk of being LBW compared with children from Biombo and Cacheu regions (Table 1). Birthweight differed by maternal age, *p* = 0.001. Children of the youngest mothers (< 20 years) weighed 2.76 kg on average, while children of the oldest mothers weighed 2.99 kg. Children of mothers with the smallest MUAC (< 240 mm) had a lower mean birthweight than mothers with larger MUAC. This was also reflected in the risk of being LBW; mothers with the smallest MUAC had higher risk of having a LBW child compared with the three quartiles with highest MUAC (Table 1). The risk of having a LBW child was similar in the three groups with highest MUAC.

**Table 1:** Association between background factors, birth weight and low birth weight (LBW)

		Background factors and low birth weight LBW < 2.500 g			Background factors and birth weight		
Background factors	Observations^a^	LBW n(%)	Prevalence ratio [95% CI]^b^	*P*-value	Mean birth weight in kg (SD)	95% CI	*P*-value^c^
Total	*N* = 628	104 (17)			2.89 (0.43)		
**Sex**				0.12			< 0.001
Boys	313	46 (15)	Reference		2.95 (0.43)	[2.90, 3.00]	
Girls	315	58 (18)	1.25 [0.94, 1.67]		2.83 (0.43)	[2.79, 2.88]	
**Twinning**				< 0.001			< 0.001
Singleton	617	98 (16)	Reference		2.90 (0.43)	[2.87, 2.93]	
Twin	11	6 (55)	3.43 [1.96, 6.03]		2.34 (0.31)	[2.15, 2.52]	
**Region**				< 0.001			0.004
Oio	347	77 (22)	Reference		2.84 (0.44)	[2.79, 2.88]	
Biombo	132	13 (10)	0.44 [0.25, 0.79]		2.96 (0.42)	[2.89, 3.03]	
Cacheu	149	14 (9)	0.42 [0.25, 0.73]		2.95 (0.39)	[2.89, 3.01]	
**Maternal BCG-scar status**^**d**^				0.70			0.92
Scar present	401	68 (17)	1.06 [0.78, 1.46]		2.89 (0.42)	[2.85, 2.93]	
No scar	226	36 (16)	Reference		2.89 (0.44)	[2.83, 2.95]	
**Socioeconomic factors**							
**Roof type**				0.57			0.45
Straw roof	134	20 (15)	Reference		2.91 (0.47)	[2.84, 2.99]	
Hard roof	492	84 (17)	1.14 [0.72, 1.83]		2.88 (0.42)	[2.85, 2.92]	
**Radio**				0.62			0.33
Yes	549	91 (17)	Reference		2.90 (0.43)	[2.86, 2.93]	
No	63	9 (14)	0.86 [0.48, 1.54]		2.85 (0.40)	[2.75, 2.95]	
**Solar panel/Generator**				0.18			0.62
Yes	280	54 (19)	1.30 [0.88, 1.93]		2.88 (0.45)	[2.83, 2.93]	
No	338	50 (15)	Reference		2.89 (0.42)	[2.85, 2.94]	
**Mobile phone**				0.01			< 0.001
Yes	369	53 (14)	Reference		2.93 (0.42)	[2.89, 2.97]	
No	232	48 (21)	1.44 [1.07, 1.93]		2.83 (0.45)	[2.77, 2.88]	
**Toilet**				0.50			0.92
No toilet	152	23 (15)	0.88 [0.62, 1.26]		2.89 (0.43)	[2.82, 2.96]	
Latrine/Toilet in the house	473	81 (17)	Reference		2.89 (0.43)	[2.85, 2.93]	
**Maternal School**				0.28			0.50
0 years of school	289	56 (19)	Reference		2.86 (0.42)	[2.82, 2.91]	
1–4 years of school	171	22 (13)	0.66 [0.40, 1.10]		2.91 (0.41)	[2.84, 2.97]	
More than 4 years of school	133	20 (15)	0.78 [0.43, 1.41]		2.92 (0.45)	[2.84, 3.00]	
**Maternal age at birth of child**				0.30			< 0.001
< 20 years	166	37 (22)	Reference		2.76 (0.41)	[2.70, 2.82]	
20–27 years	166	27 (16)	0.73 [0.43, 1.23]		2.91 (0.43)	[2.84, 2.97]	
28–35 years	162	27 (17)	0.75 [0.46, 1.23]		2.92 (0.44)	[2.86, 2.99]	
> = 36 years	132	13 (10)	0.44 [0.19, 1.02]		2.99 (0.41)	[2.92, 3.06]	
**Number of prenatal consultations**				< 0.001			0.002
None	121	34 (28)	Reference		2.79 (0.46)	[2.71, 2.87]	
1 or 2	179	27 (15)	0.54 [0.36, 0.79]		2.89 (0.39)	[2.83, 2.95]	
3 or more	271	36 (13)	0.47 [0.35, 0.63]		2.92 (0.44)	[2.87, 2.97]	
**Maternal MUAC**				< 0.001			< 0.001
1st quartile (= < 240 mm)	156	40 (26)	Reference		2.74 (0.41)	[2.68, 2.80]	
2nd quartile (242–256 mm)	159	28 (18)	0.69 [0.48, 0.99]		2.88 (0.40)	[2.82, 2.94]	
3rd quartile (258–276 mm)	149	19 (13)	0.50 [0.27, 0.91]		2.94 (0.45)	[2.87, 3.01]	
4th quartile (> = 278 mm)	150	16 (11)	0.42 [0.27, 0.64]		2.99 (0.43)	[2.92, 3.06]	
**Number of pregnancies**				0.009			0.001
1	133	33 (25)	Reference		2.76 (0.39)	[2.69, 2.82]	
2 or 3	234	31 (13)	0.53 [0.36, 0.79]		2.93 (0.41)	[2.88, 2.98]	
4 or 5	166	31 (19)	0.75 [0.44, 1.30]		2.86 (0.46)	[2.79, 2.93]	
> 5	90	9 (10)	0.40 [0.19, 0.87]		3.03 (0.43)	[2.94, 3.11]	
**Time of pregnancy registration**				0.59			0.14
1st quartile (= < 77 days)	155	27 (17)	Reference		2.88 (0.44)	[2.81, 2.95]	
2nd quartile (78–118 days)	154	23 (15)	0.86 [0.53, 1.38]		2.85 (0.39)	[2.79, 2.91]	
3rd quartile (119–152 days)	160	32 (20)	1.15 [0.76, 1.73]		2.88 (0.46)	[2.80, 2.95]	
4th quartile (> = 153 days)	158	22 (14)	0.80 [0.46, 1.40]		2.95 (0.42)	[2.89, 3.02]	
**Time of weighing**				0.86			0.34
< 24 h	193	31 (16)	Reference		2.92 (0.46)	[2.86, 2.99]	
24 - < 48 h	264	43 (16)	1.01 [0.63, 1.64]		2.86 (0.39)	[2.81, 2.91]	
48–72 h	167	30 (18)	1.12 [0.69, 1.82]		2.90 (0.45)	[2.83, 2.97]	
**Place of birth**				0.87			0.41
Home	421	70 (17)	Reference		2.89 (0.44)	[2.85, 2.93]	
Health Center	140	22 (16)	0.95 [0.62, 1.44]		2.90 (0.40)	[2.83, 2.97]	
Hospital	54	9 (17)	1.00 [0.63, 1.60]		2.93 (0.46)	[2.81, 3.05]	
Other	12	3 (25)	1.50 [0.54, 4.21]		2.73 (0.41)	[2.50, 2.96]	
**Season of birth**^**d**^				0.45			0.29
Dry Season	313	49 (16)	Reference		2.87 (0.40)	[2.83, 2.92]	
Rainy Season	315	55 (17)	1.12 [0.84, 1.48]		2.91 (0.46)	[2.86, 2.96]	

a) 628 of 633 enrolled children were weighed. Numbers do not add up to 628 due to missing information on background factors for some children

b) Standard error adjusted for cluster

c) P-value from linear regression of birthweight against the grouped background variables, corrected for intra-cluster correlation using robust standard errors

d) Rainy season: June–November; Dry season: December–May

Birthweight differed by parity (*p* = 0.001); children of mothers with a parity > 5 had a mean birthweight of 3.03 kg while firstborn children weighed on average 2.75 kg. Mothers with several prior pregnancies had lower risk of having a LBW child compared with primiparae. Children of mothers who had attended prenatal consultations had higher mean birthweight and lower risk of being LBW compared with children of mothers, who had not attended any prenatal consultations (Table 1). Not having a mobile phone was associated with higher risk of having a LBW child and lower birthweight (Table 1).

### BCG and birthweight

We did not observe a significant difference in birthweight or risk of being LBW between children of mothers with a BCG scar and children of mothers without a BCG scar (mean birthweight of 2.89 kg in children of mothers with a BCG scar and 2.89 kg in children of mothers without a BCG scar (*p* = 0.92); Prevalence ratio (PR) for LBW of 1.06 (95%CI: 0.78–1.46)) (Table 1). When including background factors one by one in the analysis only maternal age and twinning affected the estimate by more than 5%. The PR for LBW was 1.00 (95%CI: 0.72–1.37) when adjusted for maternal age while the twinning adjusted PR was 1.00 (95%CI: 0.72–1.39).

Results in Table 1 included both twins. Including only the heaviest or only the lightest twin in the birthweight analyses did not affect the conclusions (Supplementary Tables 1 and 2).

Birthweight was similar at different ages of birthweight assessment within 72 h after birth (*p* = 0.86); children weighed before 24 h had a mean weight of 2.92 kg, children weighed between 24 and 48 h had a mean weight of 2.85 kg and children weighed between 48 and 72 h had a mean weight of 2.90 kg. (Table 1).

### Determinants of BCG scar

Information on maternal BCG scar status was assessed for 1320 of the 1324 consented women. Among these, 848 (64%) mothers had a BCG scar and 472 (36%) did not have a BCG scar. Younger mothers/mothers with fewer pregnancies were more likely to have a BCG scar (*p* < 0.001) (Table 2). The distribution of background factors was similar among mothers of enrolled children (Table 2).

**Table 2:** Characteristics of women with and without a BCG scar

	BCG scar for mothers of infants enrolled in the BCGR trial^**a**^			BCG scar for all women who gave consent to enter the BCGR trial^**b**^		
	*N* = 627			*N* = 1320		
Background factors	BCG scar	No BCG scar	*P*-value^c^	BCG scar	No BCG scar	*P*-value^c^
Total	n (%) = 398 (63)	n (%) = 229 (37)		n (%) = 848 (64)	n (%) = 472 (36)	
**Region**			0.85			0.71
Oio	223 (56)	123 (54)		400 (47)	213 (45)	
Biombo	83 (21)	49 (21)		259 (31)	144 (31)	
Cacheu	92 (23)	57 (25)		189 (22)	115 (24)	
**Socioeconomic factors**^**d**^						
**Roof type**			0.22			0.12
Hard roof	319 (80)	172 (76)		660 (78)	346 (74)	
Straw roof	79 (20)	55 (24)		188 (22)	122 (26)	
**Radio**			0.52			0.86
Yes	348 (90)	200 (88)		728 (88)	412 (89)	
No	37 (10)	26 (12)		95 (12)	52 (11)	
**Solar panel/Generator**			0.47			0.25
Yes	174 (44)	105 (47)		348 (41)	178 (38)	
No	219 (56)	119 (53)		494 (59)	286 (62)	
**Mobile phone**			0.82			0.35
Yes	234 (61)	132 (60)		502 (62)	271 (59)	
No	148 (39)	87 (40)		313 (38)	188 (41)	
**Toilet**			0.99			0.20
Latrine/Toilet in the house	301 (76)	171 (76)		615 (73)	356 (76)	
No toilet	97 (24)	55 (24)		233 (27)	112 (24)	
**Maternal school**^**d**^			0.87			0.21
0 years of school	176 (46)	112 (53)		329 (41)	205 (46)	
1–4 years of school	121 (32)	50 (23)		265 (33)	121 (27)	
More than 4 years of school	83 (22)	51 (24)		216 (27)	118 (27)	
**Other background factors**						
Median maternal age at birth of child [IQR in years]^e,f^	26.1 [21.6, 30.2]	27.2 [21.5, 33]	< 0.001	25.6 [21, 30.2]	26.2 [21.1, 33.1]	< 0.001
Median number of prenatal consultations [IQR]^e,g^	2 [1, 4]	2 [1, 4]	0.19	2 [1, 4]	2 [1, 4]	0.60
Mean maternal MUAC (SD in mm)^h^	260 (26)	259 (27)	0.37	267 (31)	265 (30)	0.13
Median number of pregnancies [IQR]^e,i^	3 [2, 4]	3 [2, 5]	0.001	3 [2, 4]	3 [2, 5]	< 0.001

a) One mother of enrolled children had missing BCG scar information

b) Four consented women had missing BCG scar information

c) Distribution of background factors compared by binomial regression to express the association between scar and the background factors; intra-cluster correlation taken into account by robust standard error. Continuous variables grouped in quartiles

d) Numbers do not add up since some had missing information on background factors

e) Non-normal distributed variables presented by median and inter-quartile range (IQR)

f) Three mothers of enrolled infants and 52 women in consent cohort had missing information on maternal age

g) 57 mothers of enrolled infants and 243 women in consent cohort had missing information on number of antenatal consultations

h) MUAC: Mid upper-arm circumference; SD: Standard deviation (SD). 14 mothers of enrolled infants and 157 women in consent cohort had missing information on MUAC

i) Five mothers of enrolled infants and 12 women in consent cohort had missing information on parity

### Adverse pregnancy outcomes

One thousand three hundred twenty-four pregnant women consented to enter the BCGR trial. We obtained information on maternal BCG scar status for 1320; 848 (64%) had a BCG scar and 472 (36%) did not have a scar. Women without a BCG scar tended to have a higher overall risk of an adverse pregnancy outcome (miscarriage, stillbirth or early neonatal death prior to weighing and enrolment in the BCGR trial) compared with women with a BCG scar, PR 1.29 (95%CI 0.99–1.86), *p* = 0.06 (Table 3). Stratifying by type of pregnancy outcome revealed that women without a BCG scar tended to have higher risk of stillbirths (PR 1.55 (95%CI 0.98–2.44)) and early neonatal deaths (PR 1.51 (95%CI 0.81–2.80)) compared with women with a BCG scar, while there was little difference in risk of miscarriage, PR 0.90 (95%CI 0.50–1.63). Excluding pregnancies resulting in twin births from the analysis did not affect conclusions (Supplementary Table 3). Not having a BCG scar was thus associated with more stillbirths and early neonatal deaths, PR = 1.49 (1.07–2.08) for all pregnancies and 1.35 (0.96–1.89) for singleton births.

**Table 3:** Adverse pregnancy outcomes among all mothers who gave consent to enter the BCGR trial^a^

Maternal BCG-scar^**b**^	Total number of pregnancies^c^	Number of adverse pregnancy outcomes (%)	Prevalence Ratio [95% CI]^d^	*P*-value
All adverse pregnancy outcomes among all pregnancies^a^				0.06
**Scar**	848	82 (10)	Reference	
**No scar**	472	59 (13)	1.29 [0.99, 1.68]	
Miscarriage among all pregnancies				0.72
**Scar**	848	28 (3)	Reference	
**No scar**	472	14 (3)	0.90 [0.50, 1.63]	
Still born among all pregnancies not resulting in a miscarriage				0.06
**Scar**	820	37 (5)	Reference	
**No scar**	458	32 (7)	1.55 [0.98, 2.44]	
Early neonatal deaths among all pregnancies resulting in at least one live birth				0.20
**Scar**	784	17 (2)	Reference	
**No scar**	429	14 (3)	1.51 [0.81, 2.80]	

a) Miscarriages, still births and early neonatal deaths (death prior to enrolment in the BCGR trial)

b) 4 mothers had missing scar information

c) Including 22 twin pregnancies

d) Standard error adjusted for village cluster

Adjusting the estimated association between BCG scar and pregnancy outcome for the background factors listed in Table 2, none of the variables affected the result by more than 5%.

### Time to delivery

On average time from registration to delivery among children enrolled was similar for women with a BCG scar, 117 days, and women without a BCG scar, 114 days (*p* = 0.43).

## Discussion

### Main findings

Birthweight was assessed for 628 children in rural Guinea-Bissau among whom 67% were home born. Mean birthweight was 2.89 kg (SD 0.43) and the LBW prevalence was 17% (*n* = 104). As expected, girls had lower birthweight than boys and twins lower than singletons. We did not observe a significant difference in birthweight or risk of being LBW according to maternal BCG scar status. Women without a BCG scar tended to have higher risk of adverse pregnancy outcomes.

### Strengths and weaknesses of the study

The study was conducted within the setup of a large randomised trial conducted in the BHP rural HDSS. The clusters were initially selected using the Expanded Programme of Immunization Strategy, [24] thereby ensuring a representative sample of the population. Data collection in the rural HDSS has been ongoing since 1990. The field workers are experienced and frequently supervised. Pregnant women are followed prospectively throughout their pregnancy allowing for reliable estimates of adverse pregnancy outcomes. Registration of pregnancies was done through both two-monthly visits by the mobile teams and continuous pregnancy registration by local community key informants, thereby capturing as many pregnancies as possible. Scar information was assessed by trained BHP nurses, who were frequently supervised, ensuring high quality of the BCG scar data. A team of 15 nurses working at government health centres were trained to perform home visits shortly after birth. The nurses were trained at seminars and frequently supervised to ensure quality and uniformity. Birthweight was obtained using identical medical scales that were calibrated routinely. Therefore, we consider our weight measurements to be precise.

All children in our study were weighed within 72 h after birth, thereby ensuring data on weight in early life. Neonatal weight is known to be very dynamic, [23] however, on average the weight of children did not differ significantly by age at assessment (within the first 72 h). Due to the lack of gestational age data, we were not able to differentiate between small for gestational age and preterm birth as causes for LBW. Reliable measures of gestational age are difficult to obtain in low-resource settings, where medical equipment is scarce. Estimates of gestational age at the health centres were often based on fundal height measurements at the health centres. Available data on gestational age was based on information from the health cards and maternal recall, both of these methods can be unreliable [25]. We therefore used difference in time from registration of pregnancy to delivery as a proxy for difference in gestational length. Time between registration and delivery was similar for children of mothers with and without maternal BCG scar status.

A large number of women, who gave consent to enter the trial were not enrolled (633 children enrolled/1324 women consented). The trial was conducted in a rural setting, and it was difficult to obtain information about birth within 72 h. Giving birth is a stressful experience, especially in a low-income country where medical support for mothers giving birth at home is lacking, which is probably one of the main reasons for not informing timely about the birth of the child. Poor cell phone connection, lack of electricity to charge a cell phone, lack of a phone all together are other obstacles to timely information. We used community key informants to ensure more timely information, but they had the same phone problems, add to that travel, work or school outside the village. The enrolled cohort was similar to the consent cohort based on most measured background factors (Table 2). Thus, we consider our results to be representative.

### Consistency with other studies

We found a mean birthweight of 2.89 kg (SD 0.43) among infants in rural Guinea-Bissau. A large multi-site study spread across eight countries found a mean birthweight of 3.3 kg (SD 0.5) across all sites, [26] thus our results indicate that children in rural Guinea-Bissau on average are 400 g below the international mean according to this study. The multi-site study only measured term babies, whereas we measured birthweight of all live-born children surviving until the visit by the nurse, which may explain the difference. WHO estimates the average percentage of LBW infants in western Africa to be 15.4% with 22% for Guinea-Bissau and 64% of children not weighed [1]. Thus, our results of 17% (22% in the region of Oio) with weight obtained also for children born at home are in line with the estimates. Our results are also in line with a recent systematic review, where the LBW prevalence in Sub-Saharan Africa was 16.4% in 2000, falling to 14.0% in 2015 [5].

We used maternal MUAC during pregnancy as a measure for maternal nutritional status and found that low MUAC was associated with a lower mean birthweight and higher risk of having a LBW child, this is supported by previous studies suggesting that maternal nutritional status is an important risk factor for a child’s risk of being LBW [27–29]. In our study, we found that higher maternal age was associated with higher mean birthweight. A study of birthweight determinants in Oman from 2015 found the same association [30]. The before-mentioned multi-site study from 2017 also found that the very young women had increased odds of having a LBW child. Contrary to our findings, they found the oldest group also had an increased risk of having a LBW child [27]. In line with our results being primipara has been associated with lower birthweight and higher risk of LBW in prior studies [27, 29, 30].

### Interpretation & Implications

Birthweight data from low-income countries such as Guinea-Bissau can be difficult to obtain especially in rural areas, where many children are born at home. Estimates of birthweight among children born at home are often based on weight at the first contact with a health facility. Birthweight assessed from health cards often contain little information about when and how the child was weighed, [1] thus the birthweight estimates may be unreliable. In our study we present precise birthweight information measured in a standardised way by supervised health professionals. To our knowledge this is the first birthweight data on home-born children from rural Guinea-Bissau.

As LBW is associated with increased mortality, morbidity and bears with it high social and economic costs for a country, [1, 31–33] a high prevalence of LBW is a substantial burden for a low-income country. To target effective prevention efforts in a low-income setting, it is important to have reliable data on birthweight.

Our results suggest that women without a BCG scar may have higher risk of adverse pregnancy outcomes (Table 3), indicating that maternal BCG-vaccination might affect pregnancy outcomes. Due to the study type, we cannot conclude causation and results should be interpreted with caution. While none of our measured background factors affected the estimate by more than 5%, there may be unmeasured confounding. The results do however stress the necessity of exploring further the associations between maternal vaccination and pregnancy outcomes, both for BCG and other vaccines, especially as maternal vaccination is increasingly explored as a way of protecting children in early life.

## Conclusion

We found a mean birthweight among children in rural Guinea-Bissau of 2.89 kg (SD 0.43) and a prevalence of LBW of 17%. Several background factors were associated with birthweight and LBW. Birthweight was similar in children of mothers with and without maternal BCG scar. However, women without a BCG scar tended to experience more adverse pregnancy outcomes than women with a BCG scar, suggesting that maternal BCG-vaccination might affect perinatal health. Further studies should investigate the role of maternal immunisation on child health.

## Supplementary Information

**Additional file 1.**

## Data Availability

Data are available from the corresponding author on a collaborative basis.

## Abbreviations

BCG: Bacillus calmette-guérin vaccine
BCGR: Refers to a cluster-randomised trial entitled: “Can earlier BCG vaccination reduce early infant mortality?”
BHP: Bandim health project
CI: Confidence interval
HDSS: Health and demographic surveillance system
LBW: Low birthweight
MUAC: Mid-upper-arm circumference
NSE: Non-specific effects
OPV: Oral polio vaccine
PR: Prevalence ratio
SD: Standard deviation
TB: Tuberculosis
WHO: World health organisation
