# Supplementary material for: Factors associated with birthweight and adverse pregnancy outcomes among children in rural Guinea-Bissau - a prospective observational study
Source: BMC Public Health. 2021 Jun 17;21:1164. doi: 10.1186/s12889-021-11215-8 (PMC8212457; doi:10.1186/s12889-021-11215-8)
Supplement: Supplementary file 1 — Additional file 1. [file 12889_2021_11215_MOESM1_ESM.docx]

**Supplementary material: Factors associated with birthweight and adverse pregnancy outcomes among children in rural Guinea-Bissau. A prospective observational study.**

*Alexander Dahl Stjernholm, Sanne Marie Thysen, Igualdino Da Silva Borges, Ane Bærent Fisker*

**Supplementary Table 1: Association between background factors, birth weight and low birth weight (LBW) – including only the heaviest twin from twin pairs**

|  | | **Background factors and low birth weight** LBW < 2.500g | | |  | **Background factors and birth weight** | | |
| --- | --- | --- | --- | --- | --- | --- | --- | --- |
|  |  |  |  |  |  |  |  |  |
| **Background factors** | Observations^a^ | LBW n(%) | Prevalence ratio [95% CI]^b^ | P-value |  | Mean birth weight in kg (SD) | 95% CI | P-value^c^ |
|  |  |  |  |  |  |  |  |  |
| **Total** | N=623 | 100 (16) |  |  |  | 2.90 (0.43) |  |  |
| **Sex** | | | | 0.17 |  |  |  | <0.001 |
| Boys | 312 | 45 (14) | Reference |  |  | 2.95 (0.42) | [2.90 , 3.00] |  |
| Girls | 311 | 55 (18) | 1.23 [0.92 , 1.64] |  |  | 2.84 (0.42) | [2.79 , 2.89] |  |
| **Twinning** | | | | 0.23 |  |  |  | 0.002 |
| Singleton | 617 | 98 (16) | Reference |  |  | 2.90 (0.43) | [2.87 , 2.93] |  |
| Twin | 6 | 2 (33) | 2.10 [0.63 , 7.00] |  |  | 2.34 (0.31) | [2.15 , 2.52] |  |
| **Region** | | | | <0.001 |  |  |  | 0.002 |
| Oio | 344 | 75 (22) | Reference |  |  | 2.84 (0.44) | [2.80 , 2.89] |  |
| Biombo | 130 | 11 (8) | 0.39 [0.22 , 0.70] |  |  | 2.97 (0.42) | [2.90 , 3.04] |  |
| Cacheu | 149 | 14 (9) | 0.43 [0.25 , 0.74] |  |  | 2.95 (0.39) | [2.89 , 3.01] |  |
| **Maternal BCG-scar status^d^** |  |  |  | 0.93 |  |  |  | 0.71 |
| Scar present | 396 | 64 (16) | 1.01 [0.74 , 1.40] |  |  | 2.90 (0.42) | [2.86 , 2.94] |  |
| No scar | 226 | 36 (16) | Reference |  |  | 2.89 (0.44) | [2.83 , 2.95] |  |
| **Socioeconomic factors** |  |  |  |  |  |  |  |  |
| **Roof type** |  |  |  | 0.54 |  |  |  | 0.47 |
| Straw roof | 133 | 19 (14) | Reference |  |  | 2.92 (0.46) | [2.84 , 3.00] |  |
| Hard roof | 488 | 81 (17) | 1.16 [0.72 , 1.89] |  |  | 2.89 (0.42) | [2.85 , 2.93] |  |
| **Radio** |  |  |  | 0.70 |  |  |  | 0.27 |
| Yes | 544 | 87 (16) | Reference |  |  | 2.90 (0.43) | [2.87 , 2.94] |  |
| No | 63 | 9 (14) | 0.89 [0.50 , 1.59] |  |  | 2.85 (0.40) | [2.75 , 2.95] |  |
| **Solar panel/Generator** |  |  |  | 0.19 |  |  |  | 0.65 |
| Yes | 277 | 52 (19) | 1.31 [0.87 , 1.98] |  |  | 2.89 (0.44) | [2.83 , 2.94] |  |
| No | 336 | 48 (14) | Reference |  |  | 2.90 (0.42) | [2.85 , 2.94] |  |
| **Mobile phone** |  |  |  | 0.01 |  |  |  | <0.001 |
| Yes | 366 | 51 (14) | Reference |  |  | 2.94 (0.42) | [2.89 , 2.98] |  |
| No | 231 | 47 (20) | 1.46 [1.08 , 1.97] |  |  | 2.83 (0.44) | [2.77 , 2.89] |  |
| **Toilet** |  |  |  | 0.49 |  |  |  | 0.95 |
| No toilet | 151 | 22 (15) | 0.88 [0.60 , 1.28] |  |  | 2.90 (0.43) | [2.83 , 2.97] |  |
| Latrine/Toilet in the house | 469 | 78 (17) | Reference |  |  | 2.89 (0.43) | [2.86 , 2.93] |  |
| **Maternal School** |  |  |  | 0.29 |  |  |  | 0.55 |
| 0 years of school | 286 | 54 (19) | Reference |  |  | 2.87 (0.42) | [2.82 , 2.92] |  |
| 1-4 years of school | 170 | 21 (12) | 0.65 [0.38 , 1.11] |  |  | 2.91 (0.41) | [2.85 , 2.97] |  |
| More than 4 years of school | 133 | 20 (15) | 0.80 [0.44 , 1.45] |  |  | 2.92 (0.45) | [2.84 , 3.00] |  |
| **Maternal age at birth of child** |  |  |  | 0.32 |  |  |  | 0.003 |
| <20 years | 165 | 36 (22) | Reference |  |  | 2.76 (0.41) | [2.70 , 2.82] |  |
| 20-27 years | 165 | 26 (16) | 0.72 [0.43 , 1.21] |  |  | 2.91 (0.42) | [2.85 , 2.98] |  |
| 28-35 years | 159 | 25 (16) | 0.72 [0.43 , 1.21] |  |  | 2.94 (0.43) | [2.87 , 3.00] |  |
| >=36 years | 132 | 13 (10) | 0.45 [0.19 , 1.05] |  |  | 2.99 (0.41) | [2.92 , 3.06] |  |
| **Number of prenatal consultations** |  |  |  | <0.001 |  |  |  | 0.002 |
| None | 121 | 34 (28) | Reference |  |  | 2.79 (0.46) | [2.71 , 2.87] |  |
| 1 or 2 | 177 | 26 (15) | 0.52 [0.36 , 0.77] |  |  | 2.90 (0.39) | [2.84 , 2.95] |  |
| 3 or more | 269 | 34 (13) | 0.45 [0.33 , 0.61] |  |  | 2.93 (0.43) | [2.87 , 2.98] |  |
| **Maternal MUAC** |  |  |  | <0.001 |  |  |  | <0.001 |
| 1^st^ quartile (=<240 mm) | 155 | 39 (25) | Reference |  |  | 2.75 (0.40) | [2.68 , 2.81] |  |
| 2^nd^ quartile (242-256 mm) | 159 | 28 (18) | 0.70 [0.48 , 1.02] |  |  | 2.88 (0.40) | [2.82 , 2.94] |  |
| 3^rd^ quartile (258-276 mm) | 147 | 18 (12) | 0.49 [0.27 , 0.88] |  |  | 2.95 (0.44) | [2.88 , 3.02] |  |
| 4^th^ quartile (>=278 mm) | 148 | 14 (9) | 0.38 [0.24 , 0.59] |  |  | 3.00 (0.43) | [2.93 , 3.07] |  |
| **Number of pregnancies** |  |  |  | 0.008 |  |  |  | 0.004 |
| 1 | 133 | 33 (25) | Reference |  |  | 2.76 (0.39) | [2.69 , 2.82] |  |
| 2 or 3 | 233 | 30 (13) | 0.52 [0.35 , 0.77] |  |  | 2.93 (0.41) | [2.88 , 2.99] |  |
| 4 or 5 | 163 | 29 (18) | 0.72 [0.42 , 1.22] |  |  | 2.88 (0.46) | [2.81 , 2.95] |  |
| >5 | 89 | 8 (9) | 0.36 [0.16 , 0.80] |  |  | 3.04 (0.41) | [2.95 , 3.12] |  |
| **Time of pregnancy registration** |  |  |  | 0.45 |  |  |  | 0.10 |
| 1^st^ quartile (=<77 days) | 154 | 26 (17) | Reference |  |  | 2.89 (0.44) | [2.82 , 2.96] |  |
| 2^nd^ quartile (78-118 days) | 152 | 22 (14) | 0.86 [0.53 , 1.39] |  |  | 2.86 (0.38) | [2.80 , 2.92] |  |
| 3^rd^ quartile (119-152 days) | 160 | 32 (20) | 1.18 [0.77 , 1.81] |  |  | 2.88 (0.46) | [2.80 , 2.95] |  |
| 4^th^ quartile (>=153 days) | 156 | 20 (13) | 0.76 [0.43 , 1.33] |  |  | 2.96 (0.41) | [2.90 , 3.03] |  |
| **Time of weighing** |  |  |  | 0.86 |  |  |  | 0.38 |
| <24 hours | 192 | 30 (16) | Reference |  |  | 2.93 (0.46) | [2.86 , 2.99] |  |
| 24 - <48 hours | 261 | 41 (16) | 1.01 [0.62 , 1.63] |  |  | 2.87 (0.39) | [2.82 , 2.91] |  |
| 48 - 72 hours | 166 | 29 (17) | 1.12 [0.68 , 1.83] |  |  | 2.90 (0.45) | [2.84 , 2.97] |  |
| **Place of birth** |  |  |  | 0.99 |  |  |  | 0.41 |
| Home | 418 | 68 (16) | Reference |  |  | 2.89 (0.43) | [2.85 , 2.93] |  |
| Health Center | 139 | 21 (15) | 0.93 [0.58 , 1.49] |  |  | 2.91 (0.40) | [2.84 , 2.97] |  |
| Hospital | 54 | 9 (17) | 1.02 [0.64 , 1.64] |  |  | 2.93 (0.46) | [2.81 , 3.05] |  |
| Other | 11 | 2 (18) | 1.12 [0.28 , 4.51] |  |  | 2.77 (0.41) | [2.53 , 3.01] |  |
| **Season of birth^d^** |  |  |  | 0.60 |  |  |  | 0.29 |
| Dry Season | 312 | 48 (15) | Reference |  |  | 2.90 (0.43) | [2.87 , 2.93] |  |
| Rainy Season | 311 | 52 (17) | 1.09 [0.80 , 1.48] |  |  | 2.46 (0.33) | [2.19 , 2.73] |  |

a) Numbers do not add up to 623 due to missing information on background factors for some children.

b) Standard error adjusted for cluster.

c) P-value from linear regression of birthweight against the grouped background variables, corrected for intra-cluster correlation using robust standard errors.

d) Rainy season: June-November; Dry season: December-May.

**Supplementary Table 2: Association between background factors, birth weight and low birth weight (LBW) – including only the lightest twin from twin pairs**

|  | | **Background factors and low birth weight** LBW < 2.500g | | |  | **Background factors and birth weight** | | |
| --- | --- | --- | --- | --- | --- | --- | --- | --- |
|  |  |  |  |  |  |  |  |  |
| **Background factors** | Observations^a^ | LBW n(%) | Prevalence ratio [95% CI]^b^ | P-value |  | Mean birth weight in kg (SD) | 95% CI | P-value^c^ |
|  |  |  |  |  |  |  |  |  |
| **Total** | N=623 | 103 (17) |  |  |  | 2.89 (0.43) |  |  |
| **Sex** | | | | 0.11 |  |  |  | <0.001 |
| Boys | 310 | 45 (15) | Reference |  |  | 2.95 (0.43) | [2.90 , 3.00] |  |
| Girls | 313 | 58 (19) | 1.28 [0.94 , 1.73] |  |  | 2.83 (0.43) | [2.79 , 2.88] |  |
| **Twinning** | | | | <0.001 |  |  |  | <0.001 |
| Singleton | 617 | 98 (16) | Reference |  |  | 2.90 (0.43) | [2.87 , 2.93] |  |
| Twin | 6 | 5 (83) | 5.25 [3.44 , 8.01] |  |  | 2.34 (0.31) | [2.15 , 2.52] |  |
| **Region** | | | | <0.001 |  |  |  | 0.004 |
| Oio | 344 | 76 (22) | Reference |  |  | 2.84 (0.44) | [2.79 , 2.89] |  |
| Biombo | 130 | 13 (10) | 0.45 [0.25 , 0.81] |  |  | 2.97 (0.43) | [2.89 , 3.04] |  |
| Cacheu | 149 | 14 (9) | 0.43 [0.25 , 0.73] |  |  | 2.95 (0.39) | [2.89 , 3.01] |  |
| **Maternal BCG-scar status^d^** |  |  |  | 0.71 |  |  |  | 0.83 |
| Scar present | 396 | 67 (17) | 1.06 [0.77 , 1.46] |  |  | 2.90 (0.42) | [2.85 , 2.94] |  |
| No scar | 226 | 36 (16) | Reference |  |  | 2.89 (0.44) | [2.83 , 2.95] |  |
| **Socioeconomic factors** |  |  |  |  |  |  |  |  |
| **Roof type** |  |  |  | 0.61 |  |  |  | 0.49 |
| Straw roof | 133 | 20 (15) | Reference |  |  | 2.91 (0.47) | [2.84 , 2.99] |  |
| Hard roof | 488 | 83 (17) | 1.13 [0.71 , 1.81] |  |  | 2.89 (0.42) | [2.85 , 2.92] |  |
| **Radio** |  |  |  | 0.62 |  |  |  | 0.30 |
| Yes | 544 | 90 (17) | Reference |  |  | 2.90 (0.43) | [2.86 , 2.94] |  |
| No | 63 | 9 (14) | 0.86 [0.49 , 1.54] |  |  | 2.85 (0.40) | [2.75 , 2.95] |  |
| **Solar panel/Generator** |  |  |  | 0.14 |  |  |  | 0.61 |
| Yes | 277 | 54 (19) | 1.34 [0.91 , 1.97] |  |  | 2.88 (0.45) | [2.83 , 2.93] |  |
| No | 336 | 49 (15) | Reference |  |  | 2.90 (0.42) | [2.85 , 2.94] |  |
| **Mobile phone** |  |  |  | 0.02 |  |  |  | <0.001 |
| Yes | 366 | 53 (14) | Reference |  |  | 2.93 (0.42) | [2.89 , 2.98] |  |
| No | 231 | 47 (20) | 1.41 [1.05 , 1.87] |  |  | 2.83 (0.44) | [2.77 , 2.89] |  |
| **Toilet** |  |  |  | 0.55 |  |  |  | 0.97 |
| No toilet | 151 | 23 (15) | 0.89 [0.62 , 1.30] |  |  | 2.89 (0.43) | [2.83 , 2.96] |  |
| Latrine/Toilet in the house | 469 | 80 (17) | Reference |  |  | 2.89 (0.43) | [2.85 , 2.93] |  |
| **Maternal School** |  |  |  | 0.31 |  |  |  | 0.54 |
| 0 years of school | 286 | 55 (19) | Reference |  |  | 2.87 (0.42) | [2.82 , 2.92] |  |
| 1-4 years of school | 170 | 22 (13) | 0.67 [0.40 , 1.12] |  |  | 2.91 (0.42) | [2.84 , 2.97] |  |
| More than 4 years of school | 133 | 20 (15) | 0.78 [0.43 , 1.42] |  |  | 2.92 (0.45) | [2.84 , 3.00] |  |
| **Maternal age at birth of child** |  |  |  | 0.29 |  |  |  | <0.004 |
| <20 years | 165 | 37 (22) | Reference |  |  | 2.76 (0.41) | [2.70 , 2.82] |  |
| 20-27 years | 165 | 27 (16) | 0.73 [0.43 , 1.24] |  |  | 2.91 (0.43) | [2.84 , 2.97] |  |
| 28-35 years | 159 | 26 (16) | 0.73 [0.44 , 1.20] |  |  | 2.93 (0.44) | [2.86 , 3.00] |  |
| >=36 years | 132 | 13 (10) | 0.44 [0.19 , 1.02] |  |  | 2.99 (0.41) | [2.92 , 3.06] |  |
| **Number of prenatal consultations** |  |  |  | <0.001 |  |  |  | 0.002 |
| None | 121 | 34 (28) | Reference |  |  | 2.79 (0.46) | [2.71 , 2.87] |  |
| 1 or 2 | 177 | 26 (15) | 0.52 [0.36 , 0.77] |  |  | 2.90 (0.39) | [2.84 , 2.95] |  |
| 3 or more | 269 | 36 (13) | 0.48 [0.36 , 0.64] |  |  | 2.92 (0.44) | [2.87 , 2.97] |  |
| **Maternal MUAC** |  |  |  | <0.001 |  |  |  | <0.001 |
| 1^st^ quartile (=<240 mm) | 155 | 40 (26) | Reference |  |  | 2.74 (0.41) | [2.68 , 2.81] |  |
| 2^nd^ quartile (242-256 mm) | 159 | 28 (18) | 0.68 [0.47 , 0.98] |  |  | 2.88 (0.40) | [2.82 , 2.94] |  |
| 3^rd^ quartile (258-276 mm) | 147 | 18 (12) | 0.47 [0.27 , 0.84] |  |  | 2.95 (0.44) | [2.88 , 3.02] |  |
| 4^th^ quartile (>=278 mm) | 148 | 16 (11) | 0.42 [0.27 , 0.65] |  |  | 2.99 (0.44) | [2.92 , 3.06] |  |
| **Number of pregnancies** |  |  |  | 0.008 |  |  |  | <0.001 |
| 1 | 132 | 33 (25) | Reference |  |  | 2.76 (0.40) | [2.69 , 2.83] |  |
| 2 or 3 | 232 | 31 (13) | 0.53 [0.36 , 0.79] |  |  | 2.93 (0.41) | [2.88 , 2.99] |  |
| 4 or 5 | 165 | 31 (19) | 0.75 [0.43 , 1.31] |  |  | 2.87 (0.46) | [2.79 , 2.94] |  |
| >5 | 89 | 8 (9) | 0.36 [0.16 , 0.80] |  |  | 3.04 (0.42) | [2.95 , 3.12] |  |
| **Time of pregnancy registration** |  |  |  | 0.61 |  |  |  | 0.13 |
| 1^st^ quartile (=<77 days) | 154 | 26 (17) | Reference |  |  | 2.89 (0.44) | [2.82 , 2.96] |  |
| 2^nd^ quartile (78-118 days) | 152 | 23 (15) | 0.90 [0.56 , 1.44] |  |  | 2.85 (0.39) | [2.79 , 2.92] |  |
| 3^rd^ quartile (119-152 days) | 160 | 32 (20) | 1.18 [0.77 , 1.81] |  |  | 2.88 (0.46) | [2.80 , 2.95] |  |
| 4^th^ quartile (>=153 days) | 156 | 22 (14) | 0.84 [0.48 , 1.46] |  |  | 2.96 (0.42) | [2.89 , 3.02] |  |
| **Time of weighing** |  |  |  | 0.84 |  |  |  | 0.38 |
| <24 hours | 192 | 31 (16) | Reference |  |  | 2.92 (0.46) | [2.86 , 2.99] |  |
| 24 - <48 hours | 261 | 42 (16) | 1.00 [0.62 , 1.61] |  |  | 2.86 (0.39) | [2.82 , 2.91] |  |
| 48 - 72 hours | 166 | 30 (18) | 1.12 [0.69 , 1.82] |  |  | 2.90 (0.45) | [2.83 , 2.97] |  |
| **Place of birth** |  |  |  | 0.83 |  |  |  | 0.49 |
| Home | 418 | 69 (17) | Reference |  |  | 2.89 (0.44) | [2.85 , 2.93] |  |
| Health Center | 139 | 22 (16) | 0.96 [0.63 , 1.45] |  |  | 2.90 (0.40) | [2.83 , 2.97] |  |
| Hospital | 54 | 9 (17) | 1.01 [0.63 , 1.61] |  |  | 2.93 (0.46) | [2.81 , 3.05] |  |
| Other | 11 | 3 (27) | 1.65 [0.55 , 4.98] |  |  | 2.74 (0.43) | [2.48 , 2.99] |  |
| **Season of birth^d^** |  |  |  | 0.48 |  |  |  | 0.29 |
| Dry Season | 312 | 49 (16) | Reference |  |  | 2.90 (0.43) | [2.87 , 2.93] |  |
| Rainy Season | 311 | 54 (17) | 1.11 [0.84 , 1.46] |  |  | 2.14 (0.24) | [1.96 , 2.33] |  |

a) Numbers do not add up to 623 due to missing information on background factors for some children.

b) Standard error adjusted for cluster.

c) P-value from linear regression of birthweight against the grouped background variables, corrected for intra-cluster correlation using robust standard errors.

d) Rainy season: June-November; Dry season: December-May.

**Supplementary table 3: Adverse pregnancy outcomes among all women who gave consent to enter the BCGR trial^a^**

| Maternal BCG-scar^b^ | Total number of pregnancies | Number of adverse pregnancy outcomes (%) | Prevalence Ratio [95% CI]^c^ | P-value |
| --- | --- | --- | --- | --- |
|  |  |  |  |  |
| All adverse pregnancy outcomes among all pregnancies^a^ | | | | 0.20 |
| **Scar** | 835 | 80 (10) | Reference |  |
| **No scar** | 463 | 53 (11) | 1.19 [0.91 , 1.57] |  |
| Miscarriage among all pregnancies | | | | 0.73 |
| **Scar** | 835 | 28 (3) | Reference |  |
| **No scar** | 463 | 14 (3) | 0.90 [0.50 , 1.63] |  |
| Still born among all pregnancies not resulting in a miscarriage | | | | 0.23 |
| **Scar** | 807 | 36 (4) | Reference |  |
| **No scar** | 449 | 27 (6) | 1.35 [0.83 , 2.19] |  |
| Early neonatal deaths among all pregnancies resulting in a live birth | | | | 0.34 |
| **Scar** | 771 | 16 (2) | Reference |  |
| **No scar** | 422 | 12 (3) | 1.37 [0.72 , 2.61] |  |

1. Miscarriages, still births and early neonatal deaths (death prior to enrolment in the BCGR trial). Excluding pregnancies resulting in twin births
2. 4 mothers had missing scar information

Standard error adjusted for village cluster
